# Reduction of the spatially mutual coupling between dual-polarized patch antennas using coupled metamaterial slabs

**DOI:** 10.1038/srep30288

**Published:** 2016-07-22

**Authors:** Bai Cao Pan, Wen Xuan Tang, Mei Qing Qi, Hui Feng Ma, Zui Tao, Tie Jun Cui

**Affiliations:** 1 State Key Laboratory of Millimetre Waves, School of Information Science and Engineering, Southeast University, Nanjing 210096, China; 2 Synergetic Innovation Center of Wireless Communication Technology, Southeast University, Nanjing, 210096, China; 3 Cooperative Innovation Centre of Terahertz Science, No.4, Section 2, North Jianshe Road, Chengdu 610054, China

## Abstract

Mutual coupling inside antenna array is usually caused by two routes: signal leakage via conducting currents on the metallic background or surface wave along substrates; radio leakage received from space between antenna elements. The former one can be depressed by changing the distribution of surface currents, as reported in literatures. But when it comes to the latter one, the radiation-leakage-caused coupling, traditional approaches using circuit manipulation may be inefficient. In this article, we propose and design a new type of decoupling module, which is composed of coupled metamaterial (MTM) slabs. Two classes of MTM particles, the interdigital structure (IS) and the split-ring resonators (SRRs), are adopted to provide the first and second modulations of signal. We validate its function to reduce the radiation leakage between two dual-polarized patch antennas. A prototype is fabricated in a volume with subwavelength scale (0.6λ × 0.3λ × 0.053λ) to provide 7dB improvement for both co-polarization and cross-polarization isolations from 1.95 to 2.2 GHz. The design has good potential for wireless communication and radar systems.

The multiple-input and multiple-output (MIMO) technique, one of the most important technologies of wireless communication systems, has received tremendous attention in both academia and industry for its potential when facing the challenges of higher data rate, greater spectrum efficiency, larger average throughput and shorter latency^1^,^2^,^3^,^4^. One of the most important constituent parts of MIMO systems is the layout of each radiation element inside arrays which would directly restrict the scattering properties of the radiation components. However, the space left for antennas is usually heavily limited in the highly integrated modern communication systems. And this may lead to enhancement of mutual coupling, decrease of radiation efficiency and functional deterioration of the system, especially for those tri-layer patch antennas (with an extra reflection element) with double-polarization^5^,^6^. A series of isolation designs have been researched to overcome the coupling issues^7^,^8^,^9^,^10^,^11^,^12^,^13^,^14^,^15^,^16^,^17^,^18^,^19^,^20^,^21^,^22^,^23^,^24^,^25^,^26^,^27^,^28^,^29^,^30^,^31^,^32^,^33^. Lumped elements connected in shunt are adopted to provide a certain level of isolation between two closely arranged patch antennas^7^,^8^,^9^. A good narrow-band decoupling were achieved at 2.45 GHz with a highly compact decoupling network^9^. It is efficient but usually available in narrow-frequency band, and is too complex to be used in antenna arrays. Electromagnetic band-gap (EBG) structures are proposed between planar elements to reduce mutual coupling by restraining surface waves^6^,^10^,^11^,^12^,^13^,^14^,^15^,^16^. A dual E-shaped patch EBG has been used to improve isolation for a dual-frequency dual-polarization microstrip patch antenna^6^. And double-layer EBGs have been proposed to provide both broadband^14^ and tunable^16^ decoupling. Defected ground structures (DGS) such as different kinds of slots are etched in metallic background to improve isolation by cutting induced current^17^,^18^,^19^,^20^,^21^,^22^. It has been designed in use of broadband tri-layer patch arrays^21^ as well as dual-frequency antennas^22^. The occupied space of EBG and backward signal leakage caused by DGS are main weakness of these designs that needs to be considered. Eigen-mode decomposition method is used based on a 180° coupler^23^. Decoupling networks made of coupled resonators in multiple-element antenna arrays are also reported^24^,^25^. Such resonators could provide high-order resonance and broadband decoupling solution. Both eigen-mode and decoupling network designs are inconvenience in use of complex systems. More recently, metamaterials have been introduced for decoupling^26^,^27^,^28^,^29^,^30^,^31^,^32^,^33^.

In addition, up to now, most of the decoupling designs work on the basis of conducting current, surface wave or resonant modes. Traditional approaches with EBG and DGS structures could only handle the surface-current-type coupling and may usually occupy too much space or cause serious backward radiation. So they are inconvenient for the radio leakage problem in tri-layer microstrip patch antennas with extra reflection components. In this paper, we propose a multiple-layer metamaterial decoupling device with sub-wavelength dimensions. Aiming at all the challenges that traditional approaches have faced, the novel highly compact design is able to provide broadband isolation for radiation-leakage with dual-polarization without influencing the properties of patch antennas. The whole device consists of two sets of vertical interdigital structure (IS) particles and a 1 × 3 split-ring resonators (SRRs)) array with double-layer basement. Such design provides a significant 7 dB improvement of isolation within a relative bandwidth of 12.2% without disturbing the performance of the antennas. This design shows great potential in further decoupling system.

## Design Method

**Figure 1:**
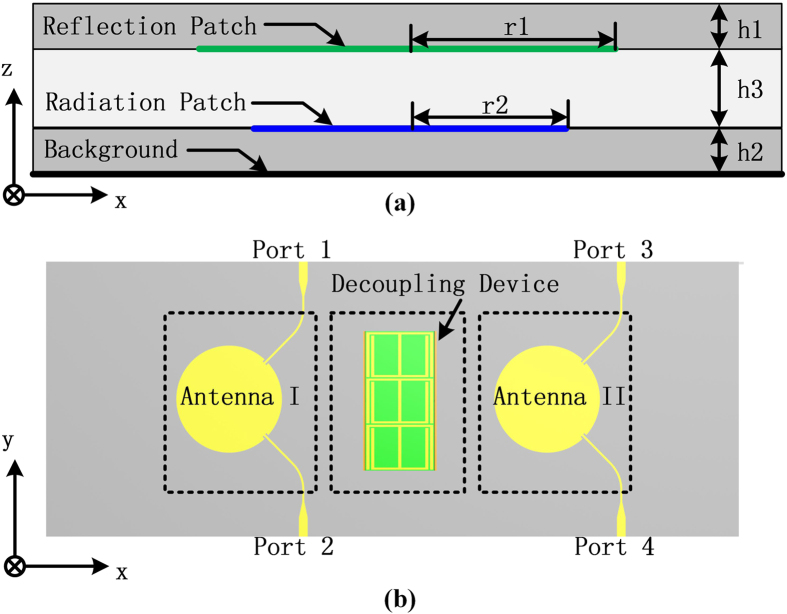
(**a**) Side view of the patch antenna. (**b**) Top view of the dual-polarized patch antenna array with decoupling design.

The design of decoupling device starts with a dual-polarized tri-layer patch antenna array which consists of the metallic background, the radiation patches and extra reflection patches. As is shown in Fig. 1(a), the circular reflection patch that is printed at the bottom of the superstrate locates right above the radiation patch with a distance of h3. The gray areas represents the superstrate and substrate and the white area is the air. The colored lines shows the position of the ground plane and the patches. All the dimensions are summarized in Table 1. Two patch antennas are arranged in a row to achieve a four-port radiating terminal, as is seen in Fig. 1(b). Two microstrip lines at port 1 and 2, each responsible for one polarization, work as excitations with an angle of ±45° from x-axis. The antenna array is supposed to work from 1.95 GHz to 2.15 GHz for communication applications. There are some concerns when we add decoupling module between the two patches. Firstly, since the patch may be fed by different excitations with orthogonal polarizations, electric field distribution would alter greatly in these two cases. Traditional EBG or DGS approaches may be workable for one certain polarization, but become incompetent to reduce mutual coupling between two adjacent patches with alterable polarizations. Secondly, the distance between two patches is electrically short at operating frequencies. Therefore, the decoupling device is supposed to be limited in width and height (usually no taller than the original patch antenna) so as not to disturb the radiation property of the antenna. Thirdly, the electric field close to the antenna plane is mainly along z-direction. To absorb radio leakage more efficiently in the limited space, we may try to re-direct the electric field and this makes it inconvenient to absorb since the illuminating area during mutual coupling is small. So the field needs to be coupled into a more convenient form.

**Figure 2:**
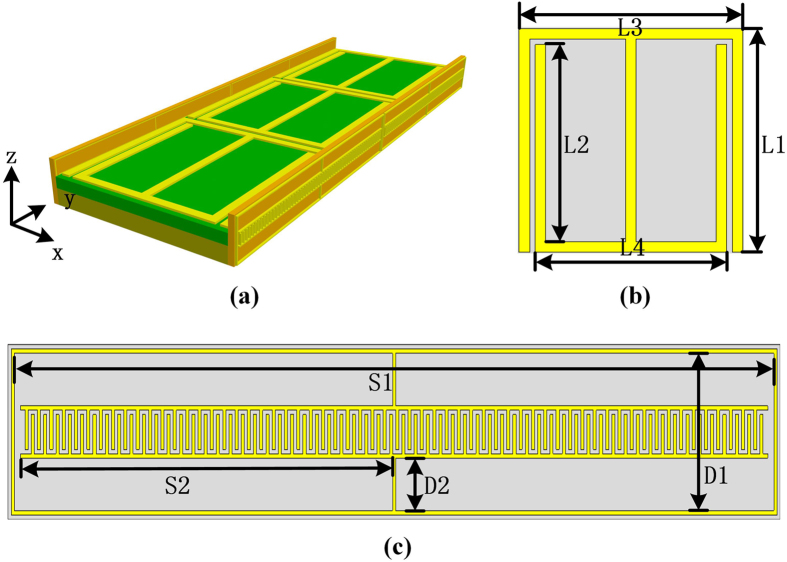
(**a**) Perspective view of the decoupling device. (**b**) The Schematic structure of SRR particle. (**c**) The schematic structure of IS particle.

Based on above considerations, a decoupling device is proposed and shown in Fig. 2. The design is composed of two rows of IS particles and a 1 × 3 array of SRR particles. The IS particles on both sides of a 100 mm × 8 mm × 1 mm substrate are in x-z plane, and SRRs on top side of a dual-layer basement is in x-y plane clamped between the ISs. Each kind of these metamaterial particles can be arranged in array to compose a “metamaterial slab” that performs a unique role for decoupling. The two IS slabs are responsible for the first-class coupling while the SRR slab is responsible for the second-class coupling. When radiated wave illuminates on the IS slabs, the electric field resonates around the metallic structure and is guided to y-direction. Then the modulated electric field causes the second-class coupling with SRRs. Instead of working at resonant mode, SRRs are designed to provide nonlinear medium property within the designed frequency band. The nonlinear-state is less efficient than the resonant-state in absorbing electromagnetic energy, however, it exists in a broader frequency band. Note that the existence of metallic background would have a strong impact on the property of SRR. Due to the boundary condition, E-field would mainly oscillate perpendicular to metallic surface and therefore the magnitude of IS-modulated field in y direction would be decreased. Hence, to avoid the guiding effect of the background, the SRR particles are lifted by a certain distance and an extra double-layer basement made of 2 mm Rogers 5880 (relative permittivity of 2.2) and 3 mm F4B (relative permittivity of 2.65) is introduced. The “metamaterial slab” can be repaired or retrofitted separately for the decoupling design. The schematic structure of SRR and ISs are exhibited in Fig. 2(b,c). The size of SRRs need to be carefully designed to avoid the resonant peaks appearing within the operating frequency band. And 1 × 3 SRR array is chosen mainly because of the limited 50 mm × 100 mm × 8 mm space. As to the IS particles, since the main effect of IS slab is to guide the radio signal into x-y plane, a small change in n-numbers of interlocking-finger in IS would not affect the properties of the whole design. And the decoupling design could be efficient as long as the n-numbers are not too small to destroy the guidance property.

Dimensions of the decoupling device are summarized in Table 2. Both the patch antenna and the decoupling device can be fabricated using printed circuit board technology at low cost.

**Figure 3:**
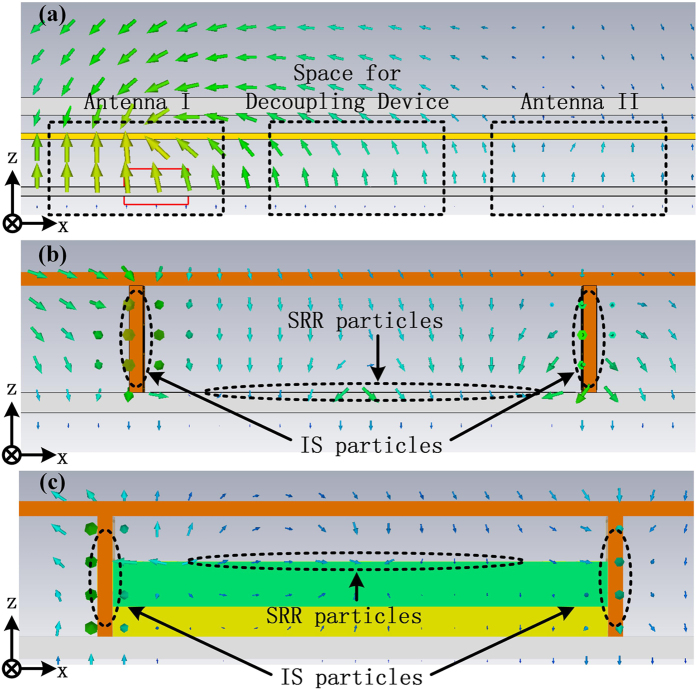
(**a**) Near E-field distribution of proposed antenna array with antenna location and space left for decoupling device marked; Near E-field distribution around decoupling device with SRRs (**b**) located on substrate directly and (**c**) lifted by a double-layer basement.

Near E-field distributions are shown in Fig. 3, in which Fig. 3(a) gives the field distribution in area of the proposed patch antenna array. The locations of antennas and the space left for decoupling device are marked. Small red rectangular gives the position of excitement for antenna I. Figure 3(b,c) give the field distributions in the space for decoupling device as is labelled in Fig. 3(a). And the location of ISs and SRRs are marked by black dashed ellipse. In Fig. 3(b), IS slabs are inserted and SRR array is printed directly on the top surface of the substrate of the patch antennas. As is seen, the field around the two IS slabs is clearly coupled into y-direction. But the field between them is still mainly along z-direction and hence SRR particles are not efficiently excited. While in Fig. 3(c), SRRs are lifted by a double-layer basement, shown by the green and yellow block. SRRs are located on the top surface of the green block. It is observed in the figure that when the basement is added, the near field changes greatly. In this way, the SRRs are excited with high-efficiency.

## Results

**Figure 4:**
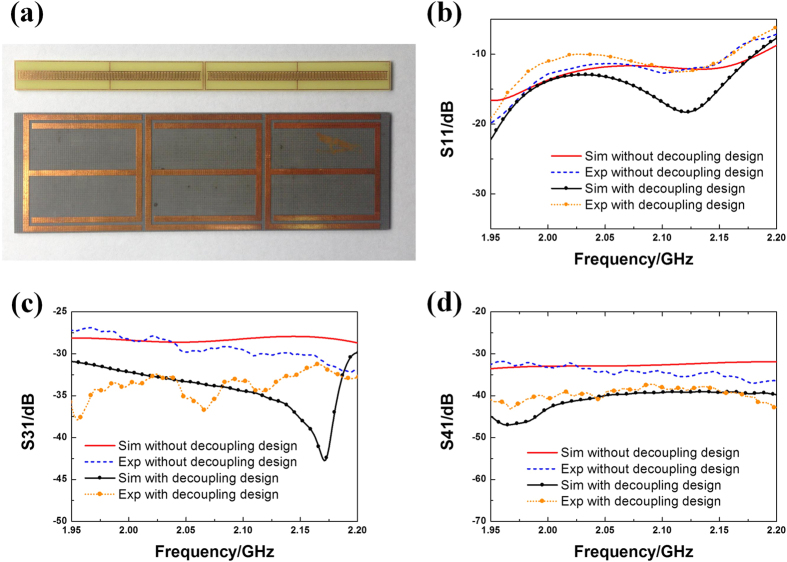
(**a**) Top view of the IS and SRR particles used in the decoupling design; Comparison of (**b**) reflection coefficients (S11), (**c**) co-polarization mutual coupling coefficients (S31) and (**d**) cross- polarization mutual coupling coefficients (S41) between simulated and measured results with and without decoupling design loaded.

Figure 4(a) exhibits the photo of the IS and SRR slabs used in the proposed decoupling device loaded between two patch antennas. The occupied space is only 0.6λ × 0.3λ × 0.053λ at central operating frequency. Experiment is carried out using Agilent Vector Network Analyzer connecting via SMA connectors and 50Ω coaxial cables. The coefficients of reflection (S11) and mutual coupling for both co-polarization (S31) and cross-polarization (S41) are measured and shown in Fig. 4(b–d). During the experiment, any ports that are not connected to Vector Network Analyzer are loaded with 50Ω broadband loads. Numerical simulations are performed in CST microwave studio as comparison. In Fig. 4(b) we observe that antennas keep efficient in the when the decoupling device is added. In experiment, S11 parameter is proved below −10 dB from 1.95 to 2.2 GHz except for a 20 MHz bandwidth at upper frequencies.

Comparison of S31 and S41 are shown in Fig. 4(c,d), respectively. It can be observed that both the co-polarization and cross-polarization mutual coupling (black lines with dots) are restrained compared with the original value (red lines). Decrement in S31 enlarges as frequency rises and an isolation improvement of 7 dB is obtained within almost the whole frequency band. Meanwhile, the isolation improvement in S41 keeps over 8 dB. The missing of significant peak at higher frequency in S31 is most probably caused by the mode-mismatching led by accuracy issues during fabrication and assembling. The measured and simulated results agree with each other well.

**Figure 5:**
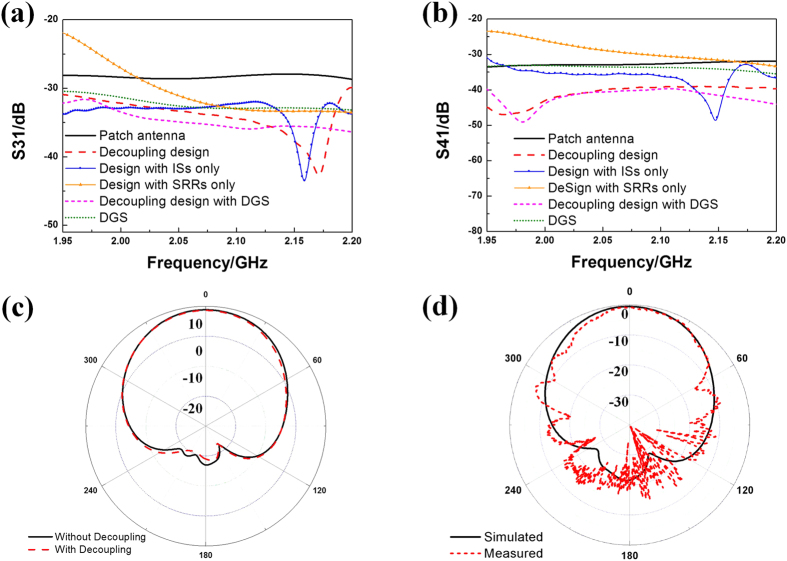
Comparison of (**a**) co-polarization mutual coupling coefficients (S31) and (**b**) cross- polarization mutual coupling coefficients (S41) between different designs. (**c**) Far-field radiation patterns of the proposed antenna array with and without decoupling design. (**d**) Simulated and measured far-field radiation patterns of antenna array with decoupling design.

Additionally, a comparison of decoupling efficiency between different designs is also shown in Fig. 5(a,b). In the figure, S31 and S41 results of antenna array with (red dash lines) and without (black lines) the decoupling design are exhibited as reference. Decoupling designs that contains IS slabs alone (blue lines with dots) or SRR slab alone (orange lines with triangles) are compared to the original design to testify the function of the proposed two-class-coupled device. The results indicate that the IS slabs provide a good co-polarization decoupling but a poor cross-polarization decoupling. On the other hand, lacking of IS slabs as secondary excitation would totally destroy the effect of SRR slab and, for this reason, the mutual coupling is rarely restrained or even enhanced at some frequencies.

In addition, owing to the double-layer basement which effectively weakens the background-effect, traditional DGS technology can be safely brought in to further improve the decoupling. Here, a pair of 35 mm × 0.4 mm y-directional slots with the separation of 5 mm is introduced. Slot-DGS is etched right below the center of SRR array to provide an extra improvement in decoupling. The results are shown by magenta short dash lines in Fig. 5(a,b). It is observed that when the Slot-DGS is added, both S31 and S41 decrease about 0.5–2 dB and becomes more constant in the operating frequency band. Results of antenna array with slot-DGS only are also shown (green short dot lines) for comparison. DGS-only-design shows a good property in decrement of S31 while S41 keeps high. From the comparison we can conclude that our design has great advantages in decoupling for both polarizations simultaneously. In the last, radiation patterns are simulated and measured (Fig. 5(c,d)). From the simulation we can see that the HPBWs of the antenna array without and with decoupling design are 73.3 and 71.4 degrees respectively while the gains are 8.8 dB and 8.6 dB, respectively. Adjunction of decoupling device does not change the radiation property of the original patch antenna. Since the position of each slab needs to be accurate, the testing environment may be a little stringent for the lab experiment. But it’s not a big problem in practical applications with outer capsulation. And the measured result agrees with the simulated one.

**Figure 6:**
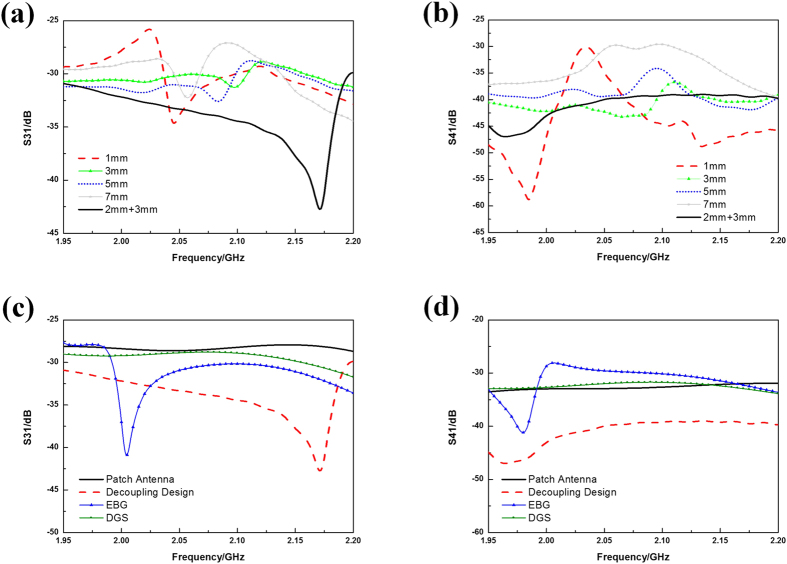
(**a**) Co-polarization mutual coupling coefficients (S31) and (**b**) cross-polarization mutual coupling coefficients (S41) between one-layer SRRs with different thicknesses of basement (changing from 1 mm to 7 mm) and the proposed double-layer SRRs with 2 + 3 mm basement (2 mm Rogers 5880 and 3 mm F4B). (**c**) Co-polarization mutual coupling coefficients (S31) and (**d**) cross-polarization mutual coupling coefficients (S41) of the patch antenna array (black lines) and that with proposed the decoupling design (red dashed lines), EBG design (blue lines with triangles) and DGS design (green lines with dots).

The influence of the thickness of the basement in SRR slab is further studied. The mutual coupling coefficients for single-layer basement with the thickness ranging from 1 mm to 7 mm and for the proposed double-layer basement are shown in Fig. 6(a,b). From the figure we can find that the co-polarization mutual coupling coefficient (S31) and the cross-polarization mutual coupling coefficient (S41) with the single-layer basement being 3 mm and 5 mm thick are more flat but are less efficient than those of the proposed design with double-layer basement. At last, the proposed decoupling design is compared with the mushroom-type EBG design and the slotted DGS design in the same patch antenna array. Since the available space is not enough for large array arrangement, the efficiency and bandwidth of EBG design are limited. And the DGS design is inefficient because the mutual coupling is mainly caused by radio leakage. Through the comparison we can briefly conclude that our design could provide efficient decoupling property for the radio leakage problem in applications like the tri-layer microstrip patch antenna arrays.

## Conclusion

A novel two-class decoupling device which manipulates radio leakage has been proposed for dual-polarization patch antenna array. The compact design is electrically-small, consisting of only two kinds of metamaterial particles, that are easy to fabricate. The mutual coupling between patches is measured to be lower than −30 dB and −40 dB for co-polarization and cross-polarization respectively. Comparison between different decoupling designs is also discussed. This low-cost design possesses a merit restraining mutual coupling within an extremely limited space, and hence has a good potential in integrated communication systems.

## Methods

Numerical simulations of the two-class decoupling device were performed by the commercial software, CST Microwave Studio. The substrate printed with the patch antenna array and IS particles was a commercial printed circuit board (F4B) with the relative permittivity 2.65 and loss tangent 0.001. The double-layer basement printed with SRR particles was a commercial printed circuit board (F4B) and a commercial printed circuit board (Rogers 5880) with the relative permittivity 2.2 and loss tangent 0.0009. In experiments, we used the Agilent vector network analyzer to measure the transmission and reflection coefficients between patch antennas and measured the far-field radiation patterns in the anechoic chamber.

## Additional Information

**How to cite this article**: Pan, B. C. *et al*. Reduction of the spatially mutual coupling between dual-polarized patch antennas using coupled metamaterial slabs. *Sci. Rep.* **6**, 30288; doi: 10.1038/srep30288 (2016).

## Figures and Tables

**Table 1:** Dimensions of the patch antenna unit: millimeters.

h1	h2	h3	R1	R2
1.5	8	1	30	22.5

**Table 2:** Dimensions of the decoupling device unit: millimeters.

L1	L2	L3	L4	S1	S2	D1	D2	w
32	28.2	32	27.4	49.6	24.1	7.6	2.4	0.5
